# Genetic and pharmacological targeting of mTORC1 in mouse models of arteriovenous malformation expose non-cell autonomous signalling in HHT

**DOI:** 10.1007/s10456-024-09961-5

**Published:** 2024-12-11

**Authors:** Antonio Queiro-Palou, Yi Jin, Lars Jakobsson

**Affiliations:** Division of Vascular Biology, Department of Medical Biochemistry and Biophysics, Karolinska Institutet, Stockholm, 171 77 Sweden

**Keywords:** Arteriovenous malformation, Endothelial, Endoglin, HHT, mTORC1, Raptor, Tsc1

## Abstract

Arteriovenous malformations (AVMs) are abnormal high flow shunts between arteries and veins with major negative impact on the cardiovascular system. Inherited loss-of-function (LOF) mutations in endoglin, encoding an endothelial cell (EC) expressed co-receptor for BMP9/10, causes the disease HHT1/Osler-Weber-Rendu, characterized by bleeding and AVMs. Here we observe increased activity of the downstream signalling complex mTORC1 within the retinal vasculature of HHT mouse models. To investigate its importance in AVM biology, concerning subvascular action, cell specificity, signalling strength and kinetics we combine timed genetic and antibody-based models of HHT with genetic mTORC1 inhibition or activation through EC specific deletion of *Rptor* or *Tsc1*. Results demonstrate that EC mTORC1 activation is secondary to endoglin LOF and mainly a consequence of systemic effects following AVM. While genetic EC inhibition of mTORC1 only showed tendencies towards reduced AVM severity, EC overactivation counterintuitively reduced it, implying that mTORC1 must be within a certain range to facilitate AVM. Complete inhibition of mTORC1 signalling by rapamycin provided the strongest therapeutic effect, pointing to potential involvement of RAPTOR-independent pathways or AVM-promoting effects of non-ECs in this pathology.

**Supplementary Information:**

The online version contains supplementary material available at 10.1007/s10456-024-09961-5.

## Introduction

Hereditary haemorrhagic telangiectasia (HHT) arises from genetic mutations leading to loss of function of either activin receptor-like kinase 1 (ACVRL1) or endoglin (ENG), which respectively act as receptors and coreceptors for bone morphogenetic proteins (BMPs) 9 and 10. The manifestation of HHT includes formation of pathological direct high-flow connections between arteries and veins, so-called arteriovenous malformations (AVMs), and recurrent bleeding often causing anaemia. In addition, AVMs lead to reduced peripheral resistance with consequences for heart function, but also to local tissue hypoxia, and if present in the lung to reduced oxygen uptake [1]. HHT patients are born with heterozygote LOF mutations but have been reported to acquire somatic LOF mutations in the corresponding wild-type (wt) allele, thereby rendering them mosaic for the expression of the respective disease-related gene/protein [2]. Mice with postnatally induced homozygote deletion of *Eng, Acvrl1*, or its downstream components *Smad4 or Smad1/5*, in a mosaic manner in ECs, present with retinal AVMs thereby representing high quality models of HHT [3–10]. In addition, genetic or antibody-based targeting of BMP-9 and 10 recapitulates the AVM phenotype [11, 12]. Previous studies show that AVM initiation – as a consequence of *Eng* deletion– associates with altered flow-induced venous to arterial EC migration, increased EC size and proliferation [5, 13]. The increase in EC proliferation in mouse models of HHT has been attributed to increased sensitivity to fluid shear stress mediated by elevated levels of KLF4 [8]. The precise contribution of individual downstream signalling components to the respective cellular feature is not fully resolved. However, we and others have described increased phosphoinositide 3-kinase (PI3K) and MAPK activity during AVM establishment in mice following induced EC-specific deletion of either *Eng* (*Eng*^*iECKO*^) or *Acvrl1* (*Acvrl1*^*iECKO*^), promoted by phosphorylation of vascular endothelial growth factor receptor (VEGFR) 2 [1, 5, 11]. Several signalling cascades, including insulin and PI3K, converge into activation of the mechanistic target of rapamycin (mTOR) kinase within the mTORC1 protein complex. mTORC1 functions as an energy and nutrient sensor that balances protein synthesis by regulation of p70-S6 Kinase 1 (S6K1) and eukaryotic initiation factor 4E binding protein 1 (4E-BP1). In accordance with PI3K activation, mTORC1 has been recorded to be activated in tissues of HHT patients as well as in HHT2 mouse models, inferred by increased levels of phosphorylated RPS6, a S6K1 target [9, 11, 12, 14, 15]. Functional relevance of the cascade in HHT pathology has been highlighted by beneficial effects of administration of the mTORC1 inhibitor rapamycin to genetic or antibody-based zebrafish- or mouse models of HHT [12, 16]. To what extent the effects are EC intrinsic, or possibly systemic and secondary to altered flow, hypoxia, inflammation or vascular permeability, have not yet been investigated. Herein we combine EC restricted gene modifications with pharmacological treatment in the mouse, to assess the impact of mTORC1 signalling in development and in progression of vascular malformation related to the disease HHT.

## Materials and methods

### Mice

*Eng*^flox/flox^ mice [17], *Tsc1*^flox/flox^ mice [18] (Tsc1^tm1Djk/J^, stock number 005680, The Jackson Laboratory) and *Rptor*^flox/flox^ [19, 20] mice (B6.Cg-Rptor^tm1.1Dmsa/J^, stock number 013188, The Jackson Laboratory) were crossed with Cdh5(PAC)-CreER^T2^ mice [21] to generate endothelial specific, tamoxifen inducible knockout mice (*Eng*^*iECKO*^, *Tsc1*^*iECKO*^, *Rptor*^*iECKO*^ and *Eng*^*iECKO*^; *Rptor*^*iECKO*^). These mice were in turn crossed with the transgenic reporter mouse line B6.Cg-Gt(ROSA)26Sortm3(CAG − EYFP)Hze/J (stock number 007903, The Jackson Laboratory) harbouring a *loxP*-flanked STOP cassette preventing transcription of a CAG promoter-driven EYFP, thereby allowing for tamoxifen-induced in vivo lineage tracing [22].

Both males and females were included. Animal housing and procedures were in accordance with Swedish legislation and approved by the local animal ethics committees.

### Tamoxifen administration

Tamoxifen (100 µg) (Sigma, T5648-5G), dissolved in corn oil (Sigma C8267), was administrated by intraperitoneal (i.p.) injection at postnatal day (P) 1 or 3 to induce gene deletion.

### Rapamycin administration

Cdh5(PAC)-CreER^T2+/-^;*Eng*^flox/flox^ or *Eng*^flox/flox^ mice were injected with tamoxifen at P3. Rapamycin (InSolution™ Rapamycin – Calbiochem; 1 mg/kg/day) or vehicle was administered at P4 and P5. Tissues were harvested at P6.

### Induction of AVMs by immunoblockade of BMP-9 and BMP-10

Cdh5(PAC)-CreER^T2^^+/-^;*Tsc1*^flox/flox^ or *Tsc1*^flox/flox^ mice were injected with tamoxifen at P1. Pups were treated with mouse monoclonal antibodies against BMP9 and BMP10 (15 mg/kg, IgG2b, MAB3209; 15 mg/kg, IgG2a, MAB2926; R&D Systems, respectively) or isotype control antibodies (15 mg/kg, IgG2b, MAB004; 15 mg/kg, IgG2a, MAB003; R&D Systems, respectively) at P3, P4 and P5 and tissues were harvested at P6.

### Whole-mount staining of the retinal vasculature

Postnatal eyes were taken and fixed for 3 h at 4 °C in 4% paraformaldehyde (PFA) in PBS, and retinas dissected. Blocking and permeabilization was done in 1% BSA and 0.5% Triton-X in PBS for 1 h at room temperature (RT). Primary antibody incubation was done in 1% BSA and 0.5% Triton-X in PBS overnight at 4 °C. Retinas were then washed at RT for 3 × 1 h with PBS/0.25% Triton-X. Secondary antibody incubation was done in 1% BSA and 0.5% Triton-X in PBS overnight at 4 °C. Retinas were then washed at RT for 3 × 1 h with PBS/0.25% Triton-X. Retinas were mounted in ProLong™ Gold Antifade Mountant (P36930, Thermofisher).

### Antibodies for immunostaining

**Table:** 

**Primary antibodies**	**Company and catalogue number**	**Dilution**
Goat anti-CD31	R&D Systems, AF3628	1/500
Rabbit anti-ERG	Abcam, ab92513	1/500
Rabbit anti- Phospho-S6 Ribosomal Protein (Ser235/236)	Cell Signaling, 2211	1/200
Rat anti-CD105	Thermofisher, 14-1051-82	1/200
Rabbit anti- Phospho-S6 Ribosomal Protein (Ser240/244)	Cell Signaling, 2215	1/200
Rat anti-CD13	Bio-Rad, MCA2183	1/200
Chicken anti-GFP	Abcam, ab13970	1/10,000
Isolectin GS-IB4 biotin-XX Conjugate	Invitrogen, I21414	1/100
Rat anti-F4/80	Abcam, ab16911	1/200
Guinea pig anti-RBPMS	Invitrogen, PA5-119676	1/500
**Secondary antibodies**	**Company and catalogue number**	**Dilution**
DyLight 405 AffiniPure Donkey Anti-Chicken IgY	Jackson Immuno, 703-475-155	1/500
Donkey anti goat Alexa Fluor^®^ 488	Invitrogen, A11055	1/500
Donkey anti-Rabbit Alexa Fluor^®^ 647	Life technologies, A31573	1/500
Donkey anti-Rat Cy3	Jackson Immuno, 712-166-153	1/500
Donkey anti-Rat Alexa Fluor^®^ 647	Jackson Immuno, 712-606-150	1/500
Donkey anti-Rabbit Alexa Fluor^®^ 555	Invitrogen, A31572	1/500
Streptavidin, Alexa Fluor™ 488 conjugate	Thermofisher, S32354	1/200
Donkey anti-Rat Alexa Fluor^®^ 647	Thermofisher, A-21,447	1/500
Donkey anti-Guinea Pig Alexa Fluor^®^ Cy3	Jackson Immuno, 706-165-148	1/500

### Microscopy

Flat-mounted retinas were imaged utilizing Zeiss Axio Observer Z1 (Carl Zeiss AG) or confocal systems LSM 700 (Carl Zeiss AG) or Leica SP8 (Leica Microsystems). For confocal acquisition the dynamic intensity range for individual channels of interests were maintained. Scanning region in Z (total imaging thickness) was set to precisely cover all vessels. Individual Z-plane thickness and step size were kept between samples for comparison.

### Image analysis and quantification

Maximum intensity projections using the ImageJ software (version 2.0.0-rc-68/1.52 h) were used for quantifications of vascular and p-RPS6 parameters.

### AVM count and AVM thickness

Average thickness of individual AVMs was calculated from the AVM area divided by its length. In AVM thickness graphs, the individual values per mouse represent average thickness from all counted shunts. AVMs were defined as vessel segments bridging an artery and vein with the thinnest diameter above 10 μm.

### Quantification of the p-RPS6^+^ area within and outside the vasculature

Manual thresholding for CD31 intensity was used to create a “vascular” region of interest (ROI) in the software ImageJ. The p-RPS6 positive areas within the CD31 + ROI were identified and divided by the CD31 + area. Individual values per mouse derive from one or two regions per retina. For assessment of p-RPS6 in the non-vascular compartment of the retina, the ROI was inverted and p-RPS6 + area was divided by the total non-vascular area.

### Quantifications of p-RPS6 in specific non-EC populations

Z-stack images of capillary beds in control mice and of AVM regions in *Eng*^*iECKO*^ mice were acquired utilizing a confocal microscope (Leica SP8, 40x objective). The number of p-RPS6 and F4/80 double positive cells within the respective frames was counted manually. Vessel area was attained from the complete CD31 signal. The number of pRPS6+, F4/80 + cells was then normalized to the vascular area for statistical analysis. For mural cell analysis, also non-AVM regions in retinas of *Eng*^*iECKO*^ mice were imaged. The number of pRPS6 + mural cells (CD13+) was counted manually for each image and normalized to maximum projection of CD31 area. Three images of different regions were analyzed and averaged for each mouse. Total ganglion cell area was measured by RBPMS + signal in maximum intensity projections. Integrated intensity of p-RPS6 staining was measured within the area and normalized to the total ganglion cell area. For each mouse, one image was analyzed.

### Image analysis for p-RPS6 in mosaic *Eng*^*iECKO*^ retinas

A selection of ENG^ +^ cells was generated by thresholding fluorescent signal from ENG staining. Intensity of p-RPS6 staining was measured within the selected area, and values were normalized to the same area (ENG^+^). Next, a selection of ENG^-^, CD31 ^+^ area was generated by clearing ENG^ +^ area from the total CD31^ +^ area. Intensity of p-RPS6 staining was measured within the selected area after applying the same thresholding method and normalized to the total ENG^-^, CD31 ^+^ area. The ratio of p-RPS6 intensity within the ENG^-^, CD31^ +^ area versus pRPS6 intensity within ENG^+^, CD31^ +^ area was calculated to evaluate the cell autonomous effect of ENG on phosphorylation of RPS6.

### Quantification of EC density

Z-stack images of fluorescently stained CD31 and ERG of leaflets of flat-mounted retinas were acquired through confocal microscopy (20X objective, Leica SP8). Maximum intensity projections were generated from z-stack images, and “vascular” ROIs were made by manual thresholding to selectively detect the CD31 + area. Analysis of number of ECs (ERG+) was made in 3D (non-projected samples), using the “3D objects counter”, within subcompartments (tip regions, veins, arteries, capillaries and AVM) of the vascular ROI.

### Quantification of vascular area

Retinas were immunolabelled for CD31 to identify the vasculature. Tile-scan images covering whole retinal vasculatures were acquired by confocal microscopy (10X objective, Leica SP8). Images were analyzed in ImageJ. Since vessels in the central part of the retinas occasionally were missing due to the dissection procedure, a circular region with a diameter of 600 μm was removed from all retinas to offset the variation from dissection. A threshold for the CD31 signal was set to cover the whole vasculature and the complete area was measured.

### Radial expansion measurement

For each sample, values of four measurements (one per quarter leaflet) of the distance from the center of the retina to the sprouting front, were averaged. To calculate the ratio, an average control radial expansion was calculated from control individuals of each individual experiment. This average control radial expansion was used as a reference to calculate the ratio.

### Image presentation and figure compilation

Adjustments of contrast and removal of irrelevant peripheral debris from dissection procedures were performed in Adobe photoshop 2024. No individual adjustments have been made in figures for which protein expression is central. Figures were compiled using Adobe Illustrator 2024.

### Statistics

Statistical analysis was done using GraphPad Prism software. Statistical significance was determined in Figures 3E and F, 5D, 6C, E and F and 7C, D and F and Suppl. Figures 1B, 1 C, 1G, 2 C and 5 C using unpaired two-tailed t-test. One-way ANOVA was used to determine statistical significance in Figures 3C, 4C, 6G, 7C and 3H and Suppl. Figures 1E, 3 C and 3D. Two-way ANOVA was used to determine statistical significance in Fig. 5H and Suppl. Figure 3B. For n, and further details see figure legends.

**Fig. 1:**
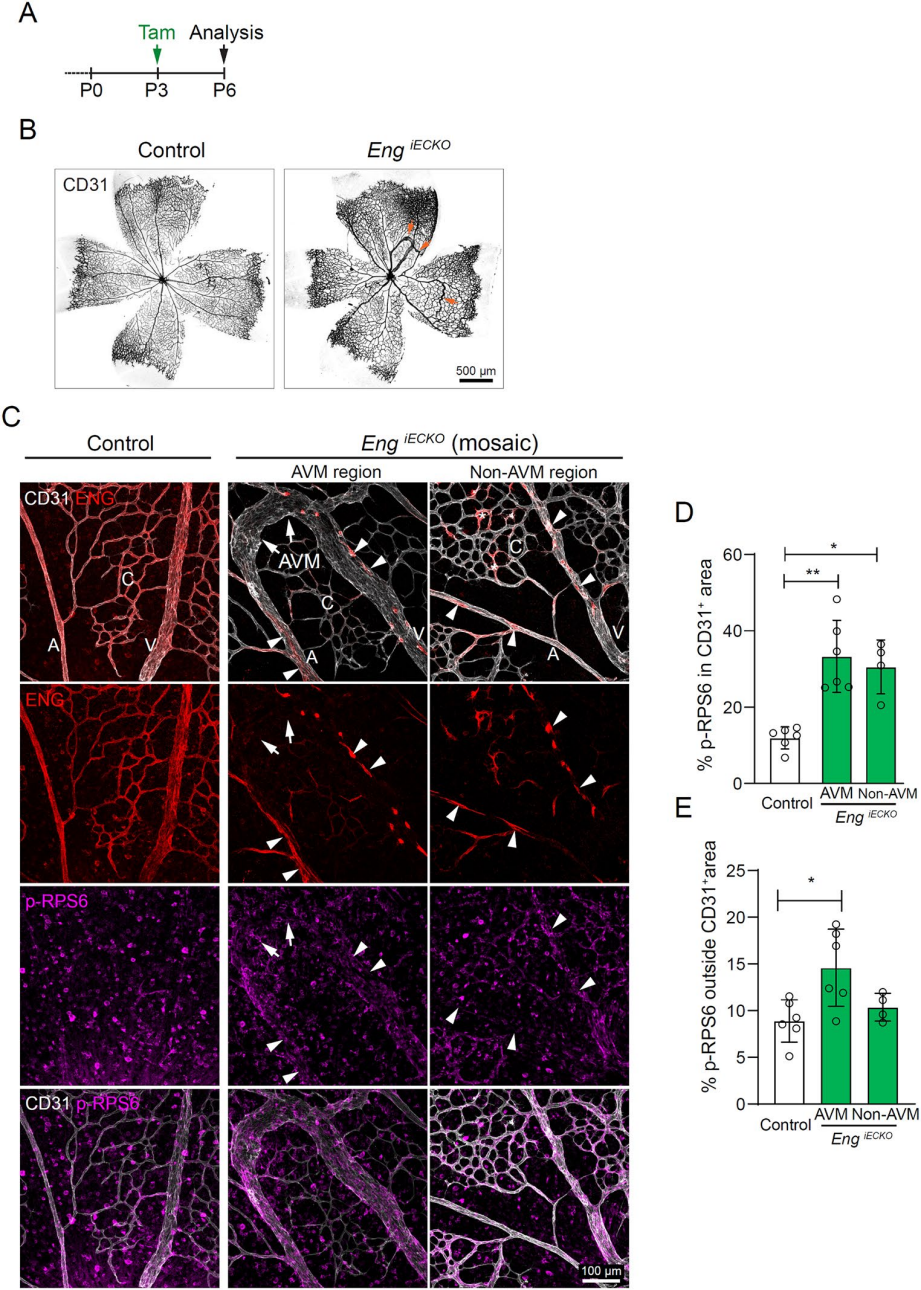
EC-specific deletion of *Eng* results in AVMs accompanied by vascular and non-vascular mTORC1 activation. **A**. Tamoxifen treatment (P3) and sample collection time point (P6) for postnatal EC deletion of *Eng* in *Eng*^*iECKO*^ mice. **B**. Retinal vasculatures of control and *Eng*^*iECKO*^ mice immunolabelled for CD31. Arrows indicate AVMs. **C**. Arteries (A), veins (V), capillaries (C) and AVMs (arrows) within central retinal regions of controls and *Eng*^*iECKO*^ mice. One AVM region and one unaffected region (non-AVM) of the same *Eng*^*iECKO*^ retina are shown. Samples were immunolabelled for CD31, ENG and p-RPS6. Arrowheads and asterisks mark non-recombined cells with retained ENG expression, indicating mosaic recombination. **D**. Quantification of the ratio of the p-RPS6 ^+^ area within the CD31^ +^ area to the complete CD31 ^+^ area, as well as the p-RPS6 ^+^ area outside the CD31 ^+^ area to the complete non-vascular area (**E**). Control (*n* = 6 mice), AVM (*n* = 6 *Eng*^*iECKO*^ mice) and non-AVM areas (*n* = 4 *Eng*^*iECKO*^ mice). Data was analysed by one-way ANOVA (Brown-Forsythe and Welch ANOVA tests). Bars indicate mean ± s.d. *p < 0.05, **p < 0.01

**Fig. 2:**
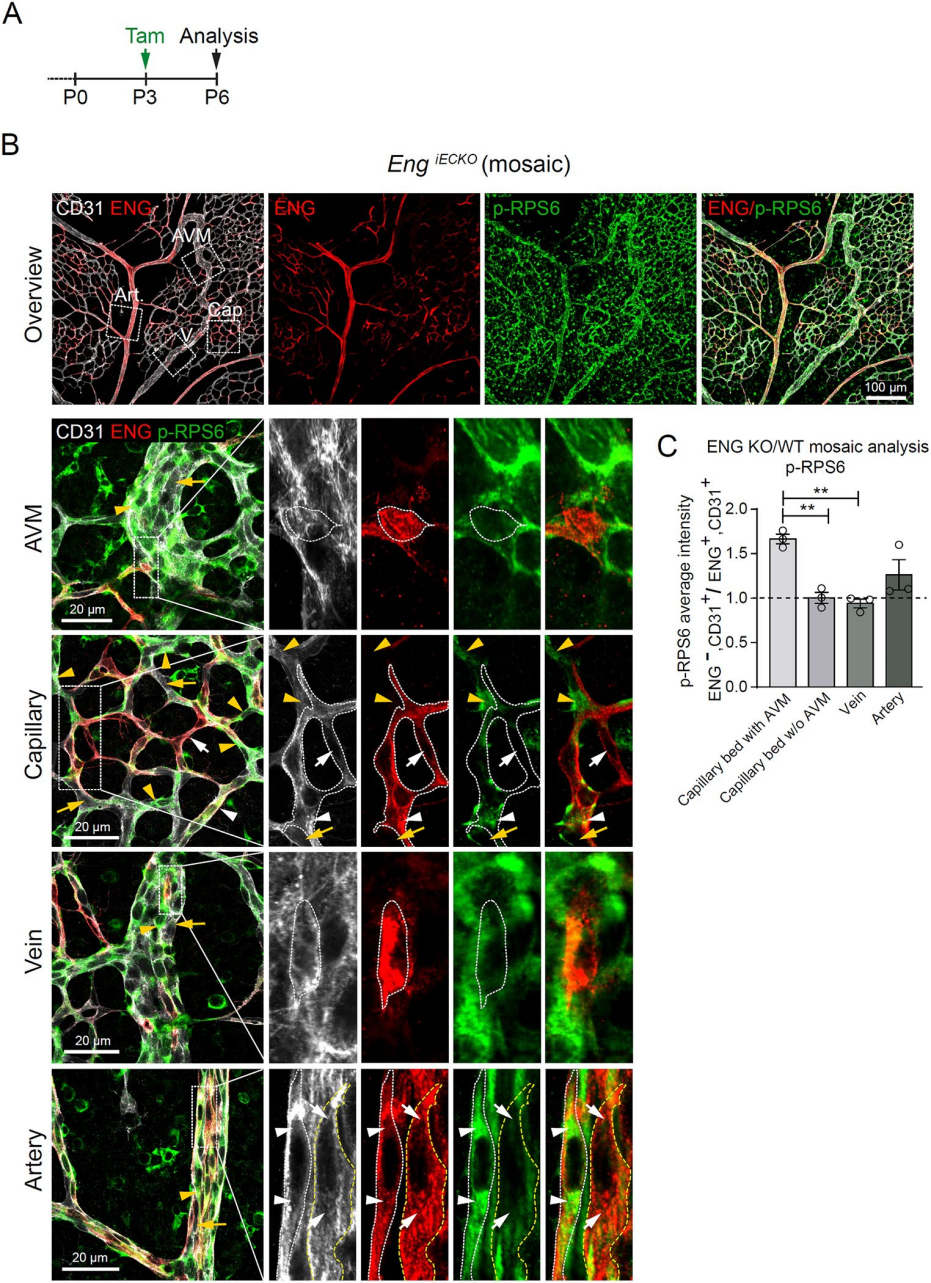
Endothelial mTORC1 activation in the malforming vasculature of *Eng*^*iECKO*^ mice is dominated by non-cell autonomous effects but shows partial cell autonomous regulation. **A**. Tamoxifen treatment (P3) and sample collection time point (P6) for postnatal EC deletion of *Eng* in *Eng*^*iECKO*^ mice. **B**. The central retinal vasculature of an *Eng*^*iECKO*^ mouse immunolabelled for CD31, ENG and p-RPS6. ENG patchiness indicates mosaic recombination. Boxed areas enclose the AVM, capillary (Cap), Vein (V) and artery (Art.) and are shown at higher magnification in lower panels. **AVM** – yellow arrowhead and arrow indicate ENG null ECs with high and low p-RPS6 levels, respectively. Magnification of boxed area indicates an individual non-recombined EC (ENG^+^) with low p-RPS6 reactivity. **Capillary** – ENG null ECs show both high (yellow arrowhead) and low (yellow arrow) p-RPS6 levels. Magnification of boxed area; example of ENG^+^ ECs with high (white arrowheads) or low (white arrow) p-RPS6 intensities. **Vein** – ENG null ECs show both high (arrowhead) and low (arrow) p-RPS6 levels. Magnification of boxed area indicates an individual non-recombined EC (ENG^+^) with low p-RPS6 reactivity. **Artery** – high variability in p-RPS6 levels between individual cells is evident. Yellow arrowhead and arrow indicate ENG null ECs with high and low p-RPS6 levels, respectively. Magnification of boxed area; ENG^+^ ECs with high (white dotted line and arrowheads) and low (yellow dotted line and white arrow) p-RPS6 intensities respectively. **C**. Quantification of the ratio of average p-RPS6 intensity in ENG^−^ CD31^+^ cells against ENG^+^ CD31^+^ in the different vascular compartments (*n* = 3 mice). Data analysed by one-way ANOVA (Brown-Forsythe and Welch ANOVA tests). Bars indicate mean ± s.d. **p < 0.01

**Fig. 3:**
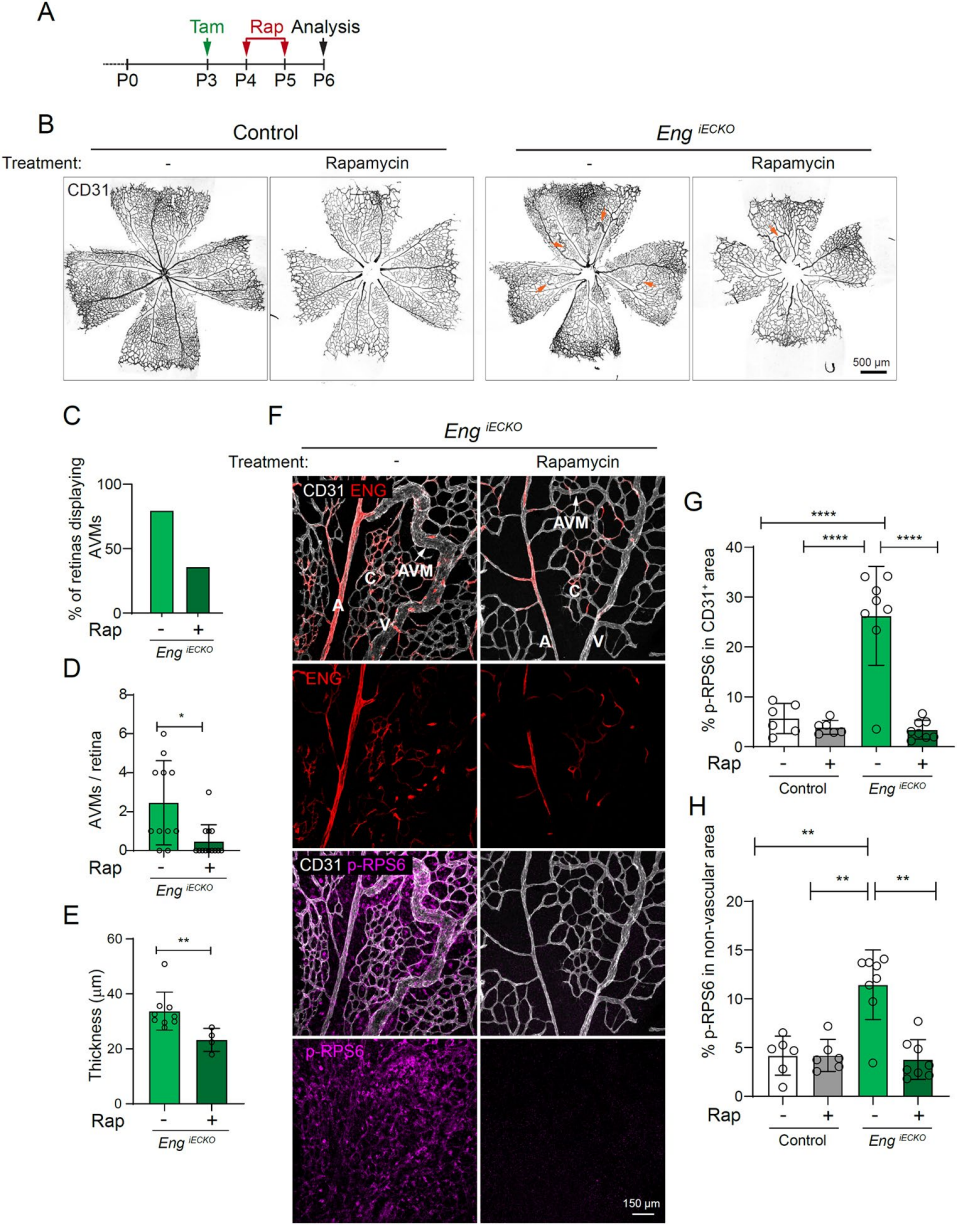
Rapamycin treatment blocks mTORC1 activity in all retinal cells and reduces AVM severity in *Eng*^*iECKO*^ mice. **A**. Tamoxifen, vehicle and rapamycin treatment and sample collection time point in control (Cre-) and *Eng*^*iECKO*^ mice. **B**. CD31 immunolabelled whole retinas of control (Cre-) and *Eng*^*iECKO*^ mice treated with vehicle or rapamycin. Arrows indicate AVMs within *Eng*^*iECKO*^ retinas. **C**-**E**. Quantification of AVM parameters in *Eng*^*iECKO*^ shows that rapamycin treatment reduces the number of retinas with at least one AVM (**C**), but also number of AVMs per retina (**D**, vehicle *n* = 11 mice, rapamycin *n* = 13 mice) and AVM thickness (**E**, vehicle *n* = 9 mice, rapamycin *n* = 4 mice). Data was analysed by two-tailed unpaired t-test with Welch’s correction. **F**. Central retinal vasculatures of *Eng*^*iECKO*^ mice treated or not with rapamycin and immunolabelled for CD31, ENG and p-RPS6. **G**. Quantification of the ratio of the p-RPS6 ^+^ area within the CD31 ^+^ area to the complete CD31^ +^ area, as well as the p-RPS6 ^+^ area outside the CD31^ +^ area to the complete non-vascular area (**H**) of different experimental groups (Control + vehicle: *n* = 6 mice; Control + rapamycin: *n* = 6 mice; *Eng*^*iECKO*^ + Vehicle: *n* = 8 mice; *Eng*^*iECKO*^ + rapamycin: *n* = 8 mice). Note that p-RPS6 area was higher also in non-vascular compartments of AVM-affected regions in untreated *Eng*^*iECKO*^ mice. Data was analysed by one-way ANOVA (Brown-Forsythe and Welch ANOVA tests). Bars indicate mean ± s.d. *p < 0.05, **p < 0.01, ****p < 0.0001

**Fig. 4:**
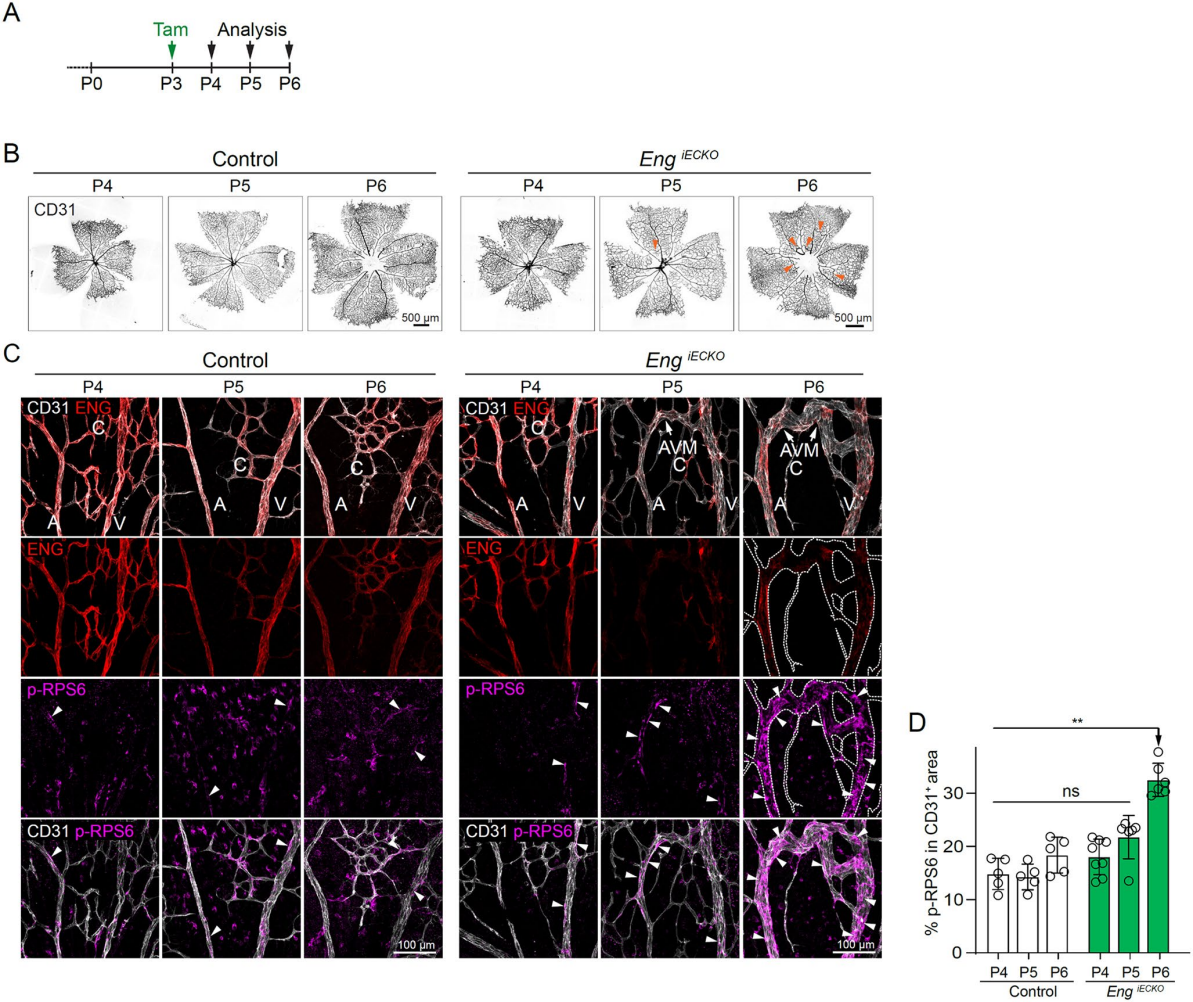
Endothelial mTORC1 activity gradually increases as a consequence of AVM establishment, not prior to it. **A**. Tamoxifen treatment (P3) and sample collection time points (P4, P5 or P6) for control and *Eng*^*iECKO*^ mice. **B**, **C**. Representative pictures of retinal vasculatures of control and *Eng*^*iECKO*^ mice, illustrating radial expansion and AVM establishment over time. **D**. Quantification of the ratio of the p-RPS6 ^+^ area within the CD31 ^+^ area to the complete CD31 ^+^ area within control retinas (white bars) as well as within *Eng*^*iECKO*^ retinas (green bars), at different postnatal days (P4 control: *n* = 5 mice; P4 *Eng*^*iECKO*^: *n* = 8 mice; P5 control: *n* = 5 mice; P5 *Eng*^*iECKO*^: *n* = 6 mice; P6 control: *n* = 5 mice; P6 *Eng*^*iECKO*^: *n* = 6 mice). Data was analysed by one-way ANOVA (Brown-Forsythe and Welch ANOVA tests). Bars indicate means ± s.d. ns, indicates that there is no statistical difference between any of the bars beneath. **p < 0.01

**Fig. 5:**
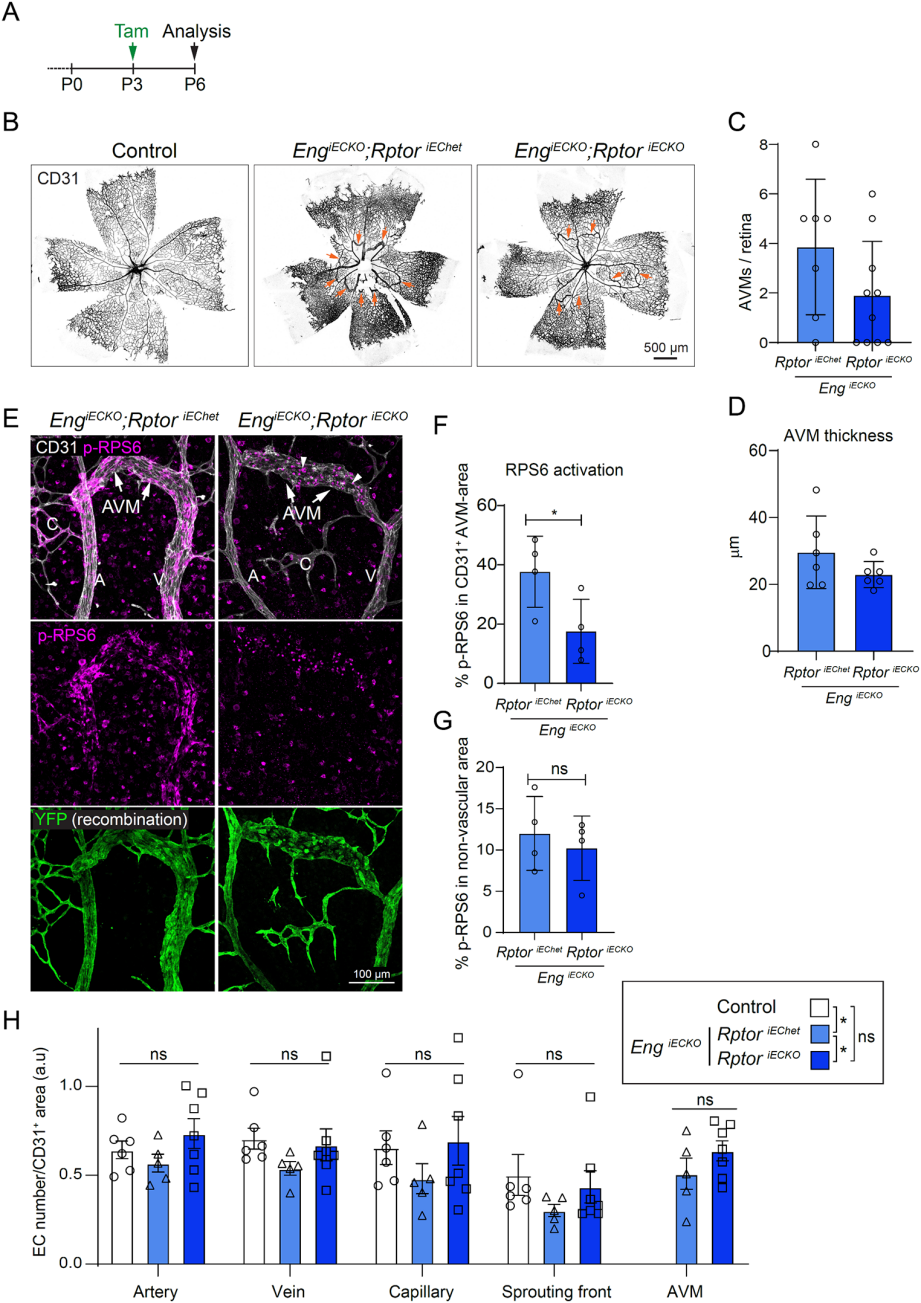
EC-specific inhibition of mTORC1 signalling has minor impact on AVM in *Eng*^*iECKO*^ mice. **A**. Tamoxifen treatment (P3) and sample collection time point (P6) for postnatal EC deletion of *Eng* and *Rptor* in *Eng*^*iECKO*^;*Rptor*^*iECKO*^ mice. **B**. Whole retinal vasculatures of control mice, *Eng*^*iECKO*^;*Rptor*^*iEChet*^ mice, and *Eng*^*iECKO*^;*Rptor*^*iECKO*^ mice immunolabelled for CD31. Arrows indicate AVMs. **C**. Quantification of numbers of AVMs/retina in *Eng*^*iECKO*^;*Rptor*^*iEChet*^ (*n* = 7 mice) and *Eng*^*iECKO*^;*Rptor*^*iECKO*^ (*n* = 10 mice) mice. **D**. AVM thickness in *Eng*^*iECKO*^;*Rptor*^*iEChet*^ (*n* = 6 mice) and *Eng*^*iECKO*^;*Rptor*^*iECKO*^ (*n* = 6 mice) mice. Data in **C** and **D** was analysed by two-tailed unpaired t-test with Welch’s correction. **E**. Central retinal vasculatures of *Eng*^*iECKO*^;*Rptor*^*iEChet*^ and *Eng*^*iECKO*^;*Rptor*^*iECKO*^ mice immunolabelled for CD31, p-RPS6, and YFP for visualization of recombination. ECs within AVMs of *Eng*^*iECKO*^;*Rptor*^*iECKO*^ mice (*n* = 4 mice) show reduced p-RPS6 abundance compared to the heterozygote control (*n* = 4 mice) (**F**), which is not true for the non-EC compartment (quantification in **G**). Data was analysed by two-tailed unpaired t-test with Welch’s correction. **H**. Quantification of the number of ERG + cells of Control (*n* = 6 mice); *Eng*^*iECKO*^; *Rptor*^*iEChet*^ (*n* = 5 mice); *Eng*^*iECKO*^; *Rptor*^*iECKO*^ (*n* = 7 mice) mice in the different vascular compartments. Note that while there is no statistically significant difference between genotypes within the subvasculatures, there are statistical differences between groups, as indicated in the upper right box. Data analysed by two-way ANOVA. Bars indicate mean ± s.d. ns, not significant; *p < 0.05

**Fig. 6:**
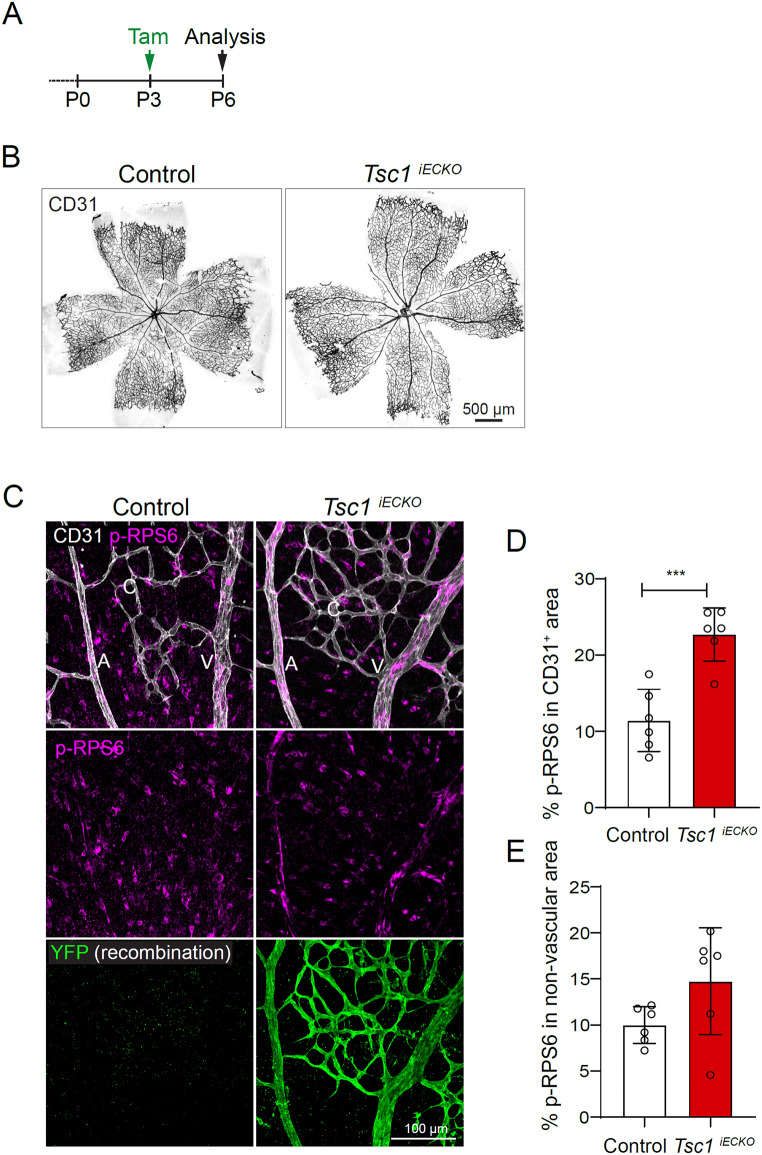
Forced EC mTORC1 activity does not provoke AVM on its own. **A**. Tamoxifen treatment (P3) and sample collection time point (P6) for postnatal EC-specific deletion of *Tsc1* in *Tsc1*^*iECKO*^ mice. **B**. CD31 immunolabelling of whole retinas of control (Cre-) and *Tsc1*^*iECKO*^ mice reveals near normal patterning following *Tsc1* deletion. **C**. Central retinal vasculatures of control (Cre-) and *Tsc1*^*iECKO*^ mice following immunolabelling for CD31, p-RPS6, and YFP (indicative of recombination). **D**. Quantification of p-RPS6 reactivity within the retinal vasculature of control and *Tsc1*^*iECKO*^ mice. Note the increased p-RPS6 reactivity specifically in ECs and not in the non-vascular compartment (**E**), comparing *Tsc1*^*iECKO*^ mice to controls, indicative of vascular specific mTORC1 gain of function. Control (*n* = 6 mice), *Tsc1*^*iECKO*^ (*n* = 6 mice). Data was analysed by two-tailed unpaired t-test with Welch’s correction. Bars indicate mean ± s.d. ***p < 0.001

**Fig. 7:**
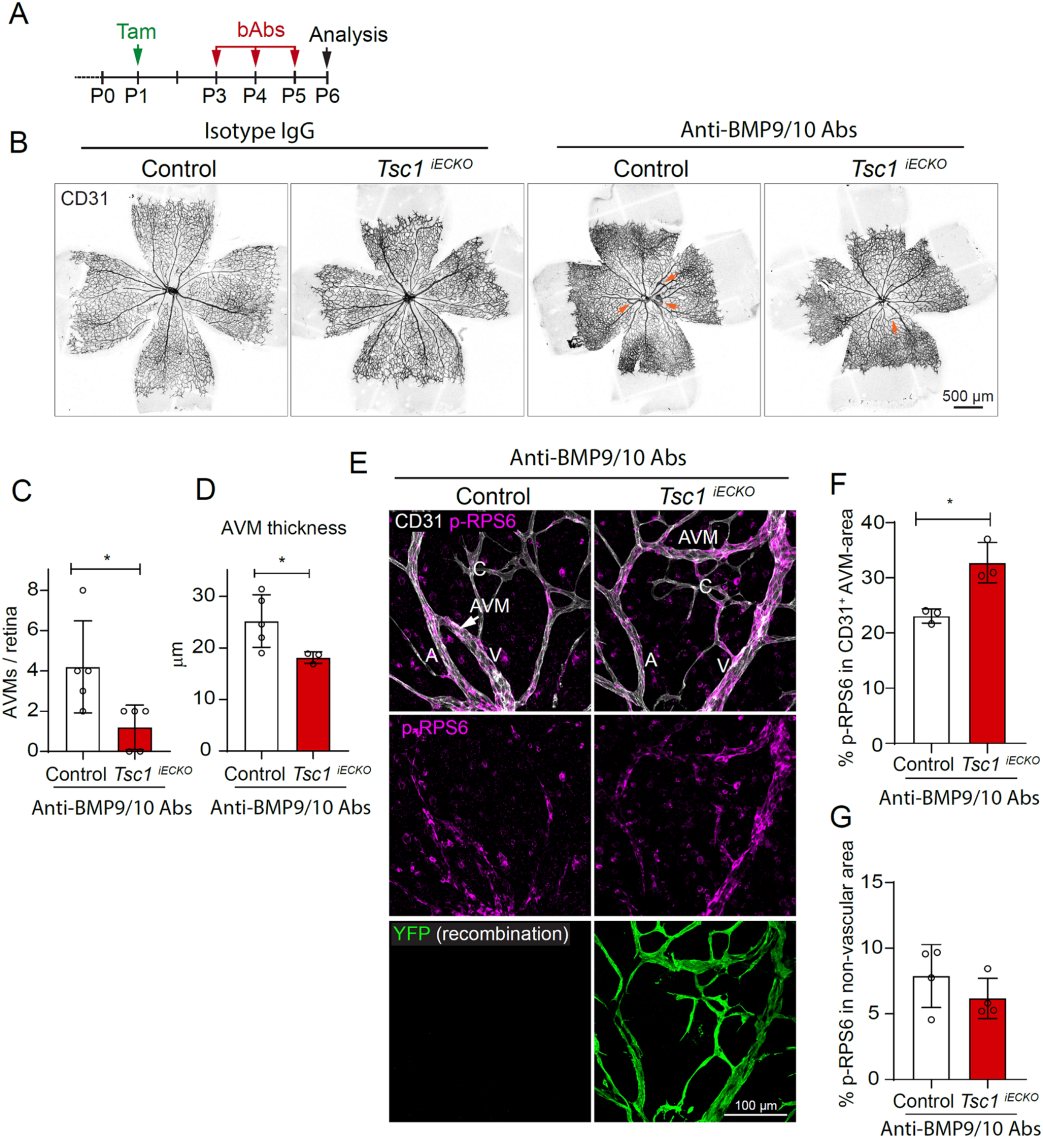
EC-specific overactivation of mTORC1 reduces incidence and thickness of AVMs induced by blocking antibodies. **A**. Time scheme for administration of tamoxifen, BMP9/10 blocking antibodies (bAbs)/Isotype IgG, and sample collection. **B**. Representative pictures showing retinal vasculatures of control (Cre-) and *Tsc1*^*iECKO*^ mice treated with IgGs or anti-BMP9/10 antibodies (Abs). Arrows indicate AVMs. **C**. Quantification of number of AVMs per retina of Control (*n* = 5 mice) and *Tsc1*^*iECKO*^ (*n* = 5 mice) mice treated with anti-BMP9/10 Abs. **D**. Quantification of AVM thickness in Control (*n* = 5 mice) and *Tsc1*^*iECKO*^ mice (*n* = 3) treated with IgGs or anti-BMP9/10 Abs. **E**. Central retinal vasculatures of control (Cre-) and *Tsc1*^*iECKO*^ mice, treated with anti-BMP9/10 Abs, immunolabelled for CD31, p-RPS6, and YFP (indicative of recombination). **F**. Quantification of the ratio of the p-RPS6 + area within the CD31 + area to the complete CD31 + area (**F**), as well as the p-RPS6 + area outside the CD31 + area to the complete non-vascular area (**G**) of central retinas of Control (*n* = 3 mice) and *Tsc1*^*iECKO*^ (*n* = 3 mice) mice treated with anti-BMP9/10 Abs. Note the vascular specific increase of p-RPS6, indicative of EC-specific mTORC1 overactivation. All data was analysed by two-tailed unpaired t-test with Welch’s correction. Bars indicate mean ± s.d. *p < 0.05

## Results

### EC-specific deletion of *Eng* results in retinal AVM accompanied by non-cell autonomous mTORC1 activation

To study effects of *Eng* deletion on AVM establishment in relation to mTOR signalling we utilised mice allowing for tamoxifen-inducible EC-specific *Eng* deletion *(Cdh5(PAC)-CreER*^*T2+/-*^:*Eng*^*flox/flox*^;*R26REYFP*, hence forth denoted *Eng*^*iECKO*^) [5]. In accordance with previous reports, tamoxifen administration at postnatal day (P) 3 resulted in retinal AVMs at P6, evidenced by confocal microscopy of flat-mounted samples stained for ECs (Fig. 1A, B) [3, 5, 23]. Immunostaining for ENG revealed uniform and abundant expression throughout the vasculature of control mice, while the vast majority ECs of *Eng*^*iECKO*^ mice had lost ENG expression (Fig. 1C). In agreement with our published data, the few remaining non-recombined ECs in *Eng*^*iECKO*^ retinas mainly localized to arteries (Fig. 1C) [5]. Note the differences in EC morphologies within arteries (elongated), veins (rounder) and AVMs (disorganized and round), exposed by individual ENG positive ECs.

Previous studies have demonstrated increased activation of the PI3K/AKT/mTORC1 signalling axis following deletion of *Alk1* (*Acvrl1*), *Eng* or its downstream component SMAD4 [9, 11, 12]. To assess potential regulation of mTORC1 downstream of ENG in vivo, we stained P6 retinas of control and *Eng*^*iECKO*^ mice for phosphorylated Serines 235/236 of RPS6 (p-RPS6), a downstream effector of mTORC1. Analysis revealed significantly increased phosphorylation of RPS6 in ECs located within AVMs but also in arteries, veins and capillaries in non-AVM regions, suggestive of direct EC-mediated effects of ENG deletion (Fig. 1C, D). At the same time, *Eng*^*iECKO*^ retinas displayed increased p-RPS6 reactivity in non-ECs surrounding AVMs but not in areas adjacent to the unaffected vasculature, compared to control retinas (Fig. 1E). Immunolabelling exposed RPS6 phosphorylation in mural cells (pericytes, smooth muscle cells, CD13^+^), a subfraction of immune cells (F4/80^+^) and ganglion cells (RBPMS+) (Suppl. Figure 1). Quantification of cells in the peri-AVM area exposed a near 2-fold increase in the number of F4/80 and p-RPS6 double-positive cells (from average 0.32 to 0.58 cells/10^3^µm^2^), but unchanged total F4/80 + area. These findings suggest that the increase in p-RPS6 derives from activation of resident microglia rather than by infiltration of peripheral macrophages, further supported by their less ramified morphology (Suppl. Figure 1 A-C). Also, an increase from 9.4% of mural cells (CD13+) being positive for p-RPS6 in controls, to 36.6% in AVM regions were observed (Suppl. Figure 1D, E). No change in ganglion cell p-RPS6 intensity was recorded (Suppl. Figure 1 F, G). Altogether, this demonstrates secondary or systemic impact on RPS6 activation on subsets of retinal cells.

To investigate potential ENG-mediated EC-autonomous regulation of mTORC1 activation, we analysed retinal vasculatures of *Eng*^*iECKO*^ mice that displayed mosaic EC-specific deletion of ENG, as a result of random incomplete genetic recombination of ECs (Fig. 2A, B). Detailed investigation of recombined (ENG^-^, CD31^+^) and non-recombined ECs (ENG^+^, CD31^+^) in close proximity to each other, exposed that ENG levels did not directly dictate RPS6 phosphorylation status, irrespective of subvascular location (AVM, capillary, vein, and artery) (Fig. 2B). Quantification of p-RPS6 intensity in ENG ^+^ versus ENG null ECs of mosaic veins, arteries as well as capillaries in non-AVM regions, showed no correlation between ENG and p-RPS6. However, in AVM-associated capillaries, ENG null ECs showed 1.7-fold higher p-RPS6 than ENG expressing ECs (Fig. 2C). This suggests a combination of ENG-mediated cell autonomous, as well as non-cell autonomous regulation of RPS6 phosphorylation exclusively in AVM-associated capillaries. At the same time the general increase in p-RPS6 in AVMs in *Eng*^*iECKO*^ mice demonstrates secondary non-cell autonomous effects – as a consequence of malformation. We further confirmed that RPS6 serines 240/244, that are specifically activated downstream of mTORC1 (and not mTORC2) [24], showed increased phosphorylation in AVMs of *Eng*^*iECKO*^ mice (Suppl. Figure 2).

### Pharmacological mTORC1 inhibition reduces retinal AVM establishment and expansion in *Eng*^*iECKO*^ mice

To assess the potential impact of mTORC1 activity on AVM establishment and expansion we administered the mTORC1 inhibitor rapamycin to control and *Eng*^*iECKO*^ mice 1- and 2-days post tamoxifen-induced recombination (see Fig. 3A for experimental outline). Rapamycin at the indicated dosage did not affect body weight, nor radial expansion of the retinal vasculature, but caused a slight reduction in the vascular area of control as well as of *Eng*^*iECKO*^ retinas (Suppl. Figure 3B, C, D). Importantly, Rapamycin reduced both AVM numbers and thickness in *Eng*^*iECKO*^ mice (Fig. 3B-E). Rapamycin efficiently inhibited mTORC1 signalling as confirmed by near complete absence of p-RPS6 in all retinal cells, including mural cells, microglia and ECs (Fig. 3F-H). The findings suggest that mTORC1 plays an important role in establishment and expansion of ENG-LOF-mediated AVM, aligning with previous reports on its effect in genetic models of HHT2 [11].

### Endothelial mTORC1 activation is recorded only after AVM initialization

Next, we studied the state of mTORC1, inferred by immunoreactivity to p-RPS6, during vascular morphogenesis in control mice and during initialization and progression of AVM in retinas of *Eng*^*iECKO*^ mice. To this end, tamoxifen was administered at P3 to either control mice or *Eng*^*iECKO*^ mice, and retinal vasculatures were analysed at different time points thereafter (Fig. 4A, B). In control mice endothelial p-RPS6 remained low and unchanged from P4-P6, with persistent expression of ENG in ECs (Fig. 4C, D). In *Eng*^*iECKO*^ mice, one day post tamoxifen administration (P4), the vasculature appeared morphologically normal and ENG remained high in most ECs, with only a fraction of ECs displaying complete loss of the ENG protein, evidenced by immunostaining for ENG. Also, the vasculature showed no recordable increase in mTORC1 activity compared to controls (Fig. 4C, D). At P5, two days post induction, ENG levels were drastically reduced, and thin AVMs appeared in the retinal vasculature, but with unchanged phosphorylation of RPS6 (Fig. 4C, D). Three days post induction of *Eng* deletion (P6), larger AVMs were evident. In agreement with data presented above, AVM-ECs at this stage displayed increased mTORC1 activity compared to all other recorded conditions in control and *Eng*^*iECKO*^ retinas (Fig. 4D). These results propose that increased EC-mTORC1-mediated S6K activity and thereby phosphorylation of RPS6, at the level detected here, plays a limited role in the process of AVM initiation, downstream of ENG-deletion.

### EC-specific inhibition of mTORC1 by *Rptor* deletion show only tendencies to reduced retinal AVM severity in *Eng*^*iECKO*^ mice

To further study the contribution of EC-derived mTORC1 to AVM development we crossed *Eng*^*iECKO*^ mice with *Rptor*^lxlx^ mice to generate EC-specific double knockouts (*Eng*^*iECKO*^;*Rptor*^*iECKO*^). *Rptor* encodes for regulatory associated protein of MTOR complex 1 (Raptor), a subunit of the mTORC1 complex required for its activity. Deletion of *Rptor* would hence result in decreased or ablated mTORC1 activity. *Eng*^*iECKO*^;*Rptor*^*iEChet*^ (Cre+, heterozygote *Rptor* deletion) and *Eng*^*iECKO*^;*Rptor*^*iECKO*^ mice induced by tamoxifen at P3, both showed retinal AVMs at P6 (Fig. 5A-B). However, *Eng*^*iECKO*^;*Rptor*^*iECKO*^ mice (homozygote *Rptor* deletion) displayed a non-significant tendency to a milder AVM phenotype than *Eng*^*iECKO*^;*Rptor*^*iEChet*^ mice, with respect to AVM incidence (number of retinas displaying at least one AVM), number of AVMs/retina and AVM thickness (Fig. 5B-D). Staining for p-RPS6 exposed reduced levels in the retinal vasculature of *Eng*^*iECKO*^;*Rptor*^*iECKO*^ mice compared to those of *Eng*^*iECKO*^;*Rptor*^*iEChet*^ mice, confirming successful EC-deletion of *Rptor* and decreased mTORC1 activity (Fig. 5E-F). However, there was no effect on p-RPS6 levels in the non-vascular compartment, indicative of EC restricted genetic manipulation (Fig. 5G). As cell size has been implicated in the biology of AVM, in particular downstream of endoglin LOF, we analysed number of ECs/subvascular area as a proxy for relative EC size. Immunostaining for the EC-restricted transcription factor ERG was used for EC counting in 3D (Suppl. Figure 4 A, B). Results did not expose any statistically significant differences in retinal EC density in arteries, veins, capillaries or sprouting front comparing control, *Eng*^*iECKO*^;*Rptor*^*iEChet*^ and *Eng*^*iECKO*^;*Rptor*^*iECKO*^ mice (Fig. 5H). Nevertheless, taking all groups into consideration, *Eng*^*iECKO*^;*Rptor*^*iEChet*^ mice showed overall statistically lower vascular EC density than both control and *Eng*^*iECKO*^;*Rptor*^*iECKO*^, whereas there was no difference on this parameter between control and *Eng*^*iECKO*^;*Rptor*^*iECKO*^ mice. These findings suggest that *Rptor* deletion partially normalises the ENG LOF-mediated increase in EC-size (Fig. 5H). EC-specific deletion of *Rptor* alone did not cause major patterning effects and did not affect EC density within the sprouting front region (Suppl. Figure 4 C-E).

These results suggest that EC-specific mTORC1 activity is not required for initiation and expansion of *Eng*-LOF mediated AVMs. Nevertheless, data indicate that the signalling cascade within ECs modestly promote AVM establishment and expansion in HHT1 mouse models. To what extent modulation of cell size, as observed by an increase following *Eng* deletion and partial normalisation by *Rptor* deletion, directly relates to the phenotype herein, remains to be investigated.

### Forced EC-specific activation of mTORC1 through induced *Tsc1* deletion reduces AVM incidence and thickness following BMP9/10 trapping

The biological relevance of the increased phosphorylation of RPS6 in ECs of HHT mouse models has not been investigated. To this end, we first generated mice (*Cdh5(PAC)-CreER*^*T2+/-*^:*Tsc1*^*flox/flox*^;*R26REYFP*, hence forth denoted *Tsc1*^*iECKO*^) allowing for induced EC-specific deletion of *Tsc1*, the endogenous inhibitor of mTORC1. Initiation of EC deletion of *Tsc1* at P3 had not caused AVMs at P6 (Fig. 6A, B). EC-specific recombination and activation of mTORC1 were confirmed by YFP expression and increased immunoreactivity to p-RPS6 (Fig. 6C, D). To investigate the effect of mTORC1 overactivation on AVM development, we first tried to establish *Eng*^*iECKO*^;*Tsc1*^*iECKO*^ double knockouts but failed due to the close proximity of their respective loci in the genome. Instead, we applied a previously published model of HHT, based on administration of BMP9 and BMP10 blocking antibodies (bAbs), to provoke AVMs within the developing retinal vasculature [11, 12]. First, tamoxifen was administered at P1 to Cre negative *Tsc1*^lxlx^ mice serving as controls, and to *Tsc1*^*iECKO*^ mice to induce endothelial *Tsc1* deletion (see Fig. 7A for administration scheme). Mice were then treated with either isotype IgG control Abs or a mix of BMP9 and BMP10 bAbs on P3, P4 and P5. Analysis at P6 revealed that all anti-BMP9/10 treated control mice had developed AVMs (Fig. 7B, C). AVMs displayed an increase of vascular p-RPS6 levels compared to IgG treated controls (Suppl. 5 A-C). However, *Tsc1*^*iECKO*^ anti-BMP9/10 treated mice showed a milder AVM-phenotype, indicated by fewer AVMs/retina and reduced AVM thickness (Fig. 7C, D). Nevertheless, endothelial mTORC1 activity was elevated in anti-BMP9/10 treated *Tsc1*^*iECKO*^ mice compared to anti-BMP9/10 treated control mice, inferred by higher immunoreactivity to p-RPS6 (Fig. 7E, F). Although severity of anti-BMP9/10-induced AVMs was reduced by TSC1 deletion, there was only a trend towards reduced non-vascular phosphorylation of RPS6 (Fig. 7G). Near complete tamoxifen-induced EC recombination was confirmed by YFP expression (Fig. 7E). These data show that forced activation of EC mTORC1 – beyond levels that naturally occur following interference with the BMP9-10/ACVRL1/ENG pathway – does not further potentiate the pathology, but instead reduces it.

## Discussion

**Fig. 8:**
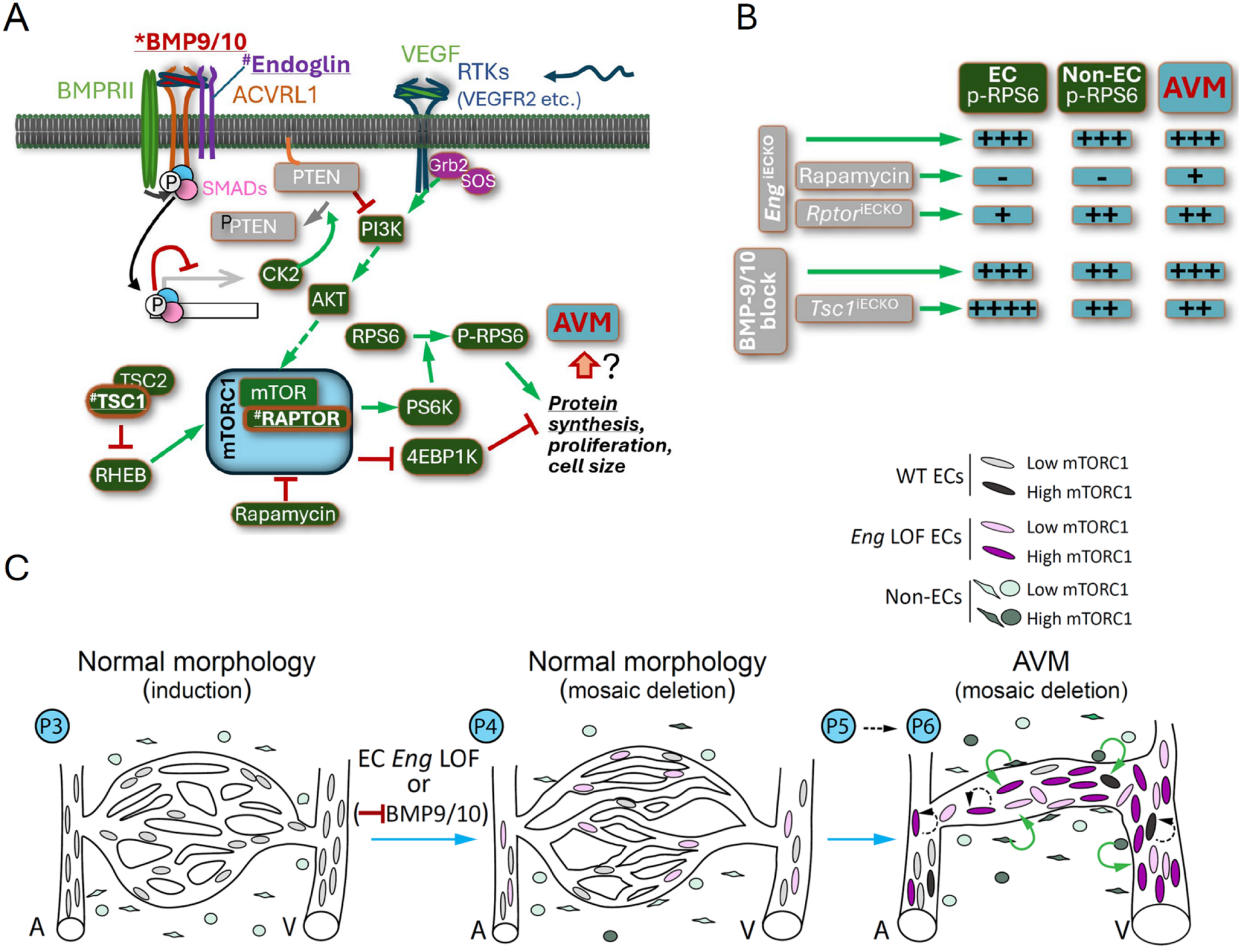
Overview of the studied signalling scheme and summary of consequences of modulation for mTORC1 and AVM status, also at the single cell level. **A**. Background to BMP9/10-ACVRL1/endoglin-SMAD signalling related to AVM in HHT. Receptor tyrosine kinases (RTK) promote PI3K activation. BMP9-SMAD indirectly inhibits PI3K by suppression of transcription of casein kinase 2 (CK2) that phosphorylates PTEN preventing it from inhibiting PI3K. PI3K indirectly activates AKT that promotes mTORC1 (blue box) activity that in turn relies on RAPTOR. mTORC1 is supressed by TSC1 and TSC2 through their inhibitory action on RHEB that acts as an mTORC1 stimulator. mTORC1 activates S6K that phosphorylates RPS6 that promotes protein synthesis. mTORC1 also inhibits 4EBP1K that normally inhibits protein synthesis. Herein we have utilized EC-specific inducible deletion of *Eng*, *Rptor*, and *Tsc1*, as well as applied blocking Abs to BMP9/10 and inhibition of the mTORC1 by the drug Rapamycin, in the mouse. **B**. Summary of effects of genetic-, drug- or antibody-based modulation with respect to mTORC1 activation in ECs and non-ECs, as well as on AVM status. **C**. Illustration of the retinal capillary bed of *Eng*^*iECKO*^ mice from P3 (day of induction of gene deletion) to P6. Oval shapes within vessels represent ECs (either *Eng* LOF-pink, or WT-grey) and round and irregular objects outside of vessels represent non-ECs (green). Light and dark colours indicate low and high levels of RPS6 phosphorylation, respectively. At P3, just after induction, ECs and non-ECs of the central vasculature generally have low levels of mTORC1. At P4, the vasculature is structurally unchanged despite full loss of ENG in subpopulations of ECs. At P5 (not shown), minor AVMs are observed, with a trend of increased mTORC1 (Fig. 4C, D) within ECs. At P6, mTORC1 is high in ECs within AVMs but also in normal vascular regions within the same retina. This is not related to the ENG levels of individual ECs suggestive of secondary affects. In addition, also non-ECs (i.e. microglia and mural cells) of AVM regions show increased mTORC1 compared to control retinas. The fact that EC-specific genetic inhibition (*Rptor* deletion) has much less potent effects on AVM than rapamycin suggests that mTORC1 of non-ECs plays a major role in this pathology, likely by paracrine actions on the endothelium (green circular arrows). Data also suggests that mTORC1 in ECs themselves plays a limited role in the pathology (dashed black circular arrows)

Prior studies have demonstrated that genetic or pharmacological inhibition of the BMP9-10/ACVRL1/ENG/SMAD1,5 cascade results in increased AKT activity, mediated by various co-stimulatory factors, including VEGF [5, 9, 11]. AKT is also regulated by fluid shear stress, providing a challenge in resolving cell-autonomous versus non-cell autonomous action during active morphogenesis [8]. In vivo observations of direct increased AKT activity in ECs upon inhibition of this cascade are however missing. Activation of AKT has instead been inferred from recorded phosphorylation of RPS6 – a substrate of S6 Kinase exclusively activated by mTORC1 (See Fig. 8A for schematic overview of the signalling pathway). Although these cascades are known to be connected, mTORC1 activity is not exclusively regulated by AKT which in turn has several downstream targets. In this study we focus solely on the specific contribution of mTORC1 to the establishment and expansion of AVMs in HHT-related biology. The mTOR inhibitor rapamycin has shown promise in HHT therapy, but the cellular mode of action responsible for its effect has not been clarified. Herein we noticed that systemic inhibition of mTOR by administration of rapamycin, reduced both frequency and size of AVMs in *Eng*^*iECKO*^ mice. In contrast, genetically induced inhibition of mTORC1 specifically in ECs had only mild beneficial effect on the AVM phenotype (Fig. 8B). This discrepancy points to that mTORC1 activity in non-ECs significantly contributes to the AVM phenotype. Interleukin-1β, tumor necrosis factor α (TNF α) and interferon-γ have been shown to induce microglial phosphorylation of RPS6 [25]. Hence, activation of RPS6 in peri-AVM microglia of *Eng*^*iECKO*^ retinas may indicate the presence of inflammatory cytokines that in turn would cause vascular destabilisation. TNFa has indeed been shown to provoke vascular shunting in yolk sacs of mice with EC-specific heterozygosity for Smad 1 and 5, which alone did not develop such malformations [26]. Furthermore, the increase in p-RPS6 within the AVM-associated mural cell population demonstrates tissue effects, accompanying AVM progression. The precise potential impact of increased microglial and mural cell mTORC1 activity on paracrine signalling and on ENG LOF-mediated AVM remains unknown. Along with previously published observations it is tempting to propose that paracrine signalling of inflammatory cytokines on the endothelium may be instrumental. Even though Rapamycin at the concentration applied in this study did not affect weight gain of pups, the recorded reduction in retinal vascular area calls for caution in dosage. While inducible cell-restricted gene deletion can be utilized to dissect complex biological questions, it requires deep understanding of recombination rates. Herein, we use inducible reporter constructs in combination with staining for the targeted ENG protein, thereby providing detailed information on functional recombination rates. Efficient deletion of RAPTOR was in addition confirmed by deep analysis of individual z-stacks confirming near complete inhibition of EC-restricted p-RPS6 following EC-specific deletion of *Rptor* (not shown). The difference in AVM-phenotypic outcome between drug and genetic targeting of mTORC1 is therefore unlikely a consequence of differential EC-specific mTORC1 inhibition, although it cannot be fully ruled out. Although EC restricted deletion of *Eng* is sufficient to initiate AVM within the developing retina, the findings highlight the multicellular aspect in the progression of this disease.

Even though prior studies have indicated alterations in AKT activation following manipulation of ACVRL1 and ENG in ECs in vitro, mosaic analysis and gene deletion herein did not confirm a direct correlation between ENG levels and p-RPS6 in vivo. Observations of p-RPS6 levels from early ENG deletion through AVM progression demonstrated an increase in phosphorylation of RPS6 only after vessel morphology was changed (Fig. 8C), suggesting the phenomena to be mainly secondary to tissue alterations [8, 27]. Secondary drivers may include altered blood flow, which has been shown to affect the cascade [28, 29]. However, ECs within regions of normal vasculature, in retinas displaying AVMs in other regions, still showed increased mTORC1 activity suggestive of additional causes, such as regional hypoxia or paracrine factors including ANG2 [30, 31].

The process of AVM in HHT is known to involve defective EC fluid shear responses, migratory behaviour, cell size and cell cycle regulation but what cellular properties that are affected by mTORC1 that in turn contribute to disease progression, are not understood. The mTORC1 protein complex plays central roles in metabolism, protein synthesis, and thereby cellular growth and proliferation of many cell types. A report by Ouarné and colleagues describes increased EC size in a non-genetic mouse model of retinal arterio-venous shunts. Treatment with the mTORC1 inhibitor everolimus, reduced and reverted shunting, accompanied by reduced cell size [32]. In our study, the slight increase in average EC-size of *Eng*^*iECKO*^ mice was normalised by EC-specific deletion of *Rptor*, indicating that such effects may be EC intrinsic. Further studies are required to settle the dominating impact of the cascade on AVM biology [33–36].

To increase activity of mTORC1 we conditionally deleted TSC1, an endogenous inhibitor of mTORC1, in the endothelium. Although the expression level of *Tsc1* in ECs is low (data not shown), deletion of endothelial *Tsc1* caused an increase in p-RPS6, an increase that on its own had no significant effect on vascular parameters assessed here. The fact that removal of this endogenous suppressor causes higher activity of its target, certifies the presence of upstream basal activity. Despite higher endothelial mTORC1 activity within AVMs following antibody-based blocking of BMP9 and 10 in these mice, AVMs showed reduced thickness, compared to AVMs in control mice. Although the precise cellular behaviour underlying the reduced AVM severity is unclear, it may relate to reduced proliferation, a phenomenon reported in other cells following *Tsc1* deletion [37]. *Tsc1* deletion has been shown to cause partial suppression of mTORC2 activity and thereby reduced AKT phosphorylation, which may reduce progression through the cell cycle [38]. These data suggest a complex integration of endothelial mTORC1 signalling in vascular patterning related to vascular anomalies and clearly demonstrate that the pathway is not deterministic in AVM biology.

The BMP9,10/ACVRL1/ENG/SMAD pathway has shown to impact EC metabolism. To what degree these effects rely on mTOR signalling, known to play a central role in metabolism across various cell types, is an interesting question for long term maintenance of the vascular system that also feeds into cell cycle regulation, a central component in HHT pathology [39].

In conclusion the data indicate that neither establishment nor expansion of AVMs requires mTORC1 signalling within the endothelium itself. These findings question the strategy of endothelial cell-specific targeting of mTOR in HHT but speaks in favour of systemic mTORC1 targeting in combinatorial treatment.

## Electronic supplementary material

Below is the link to the electronic supplementary material.

Supplementary Material 1

## Data Availability

Data is provided within the manuscript or supplementary information files.
