# Supplementary material for: Genetic and pharmacological targeting of mTORC1 in mouse models of arteriovenous malformation expose non-cell autonomous signalling in HHT
Source: Angiogenesis. 2024 Dec 11;28(1):6. doi: 10.1007/s10456-024-09961-5 (PMC11634917; doi:10.1007/s10456-024-09961-5)
Supplement: Supplementary file 1 — Supplementary Material 1 [file 10456_2024_9961_MOESM1_ESM.pdf]

Supplementary Figure 1

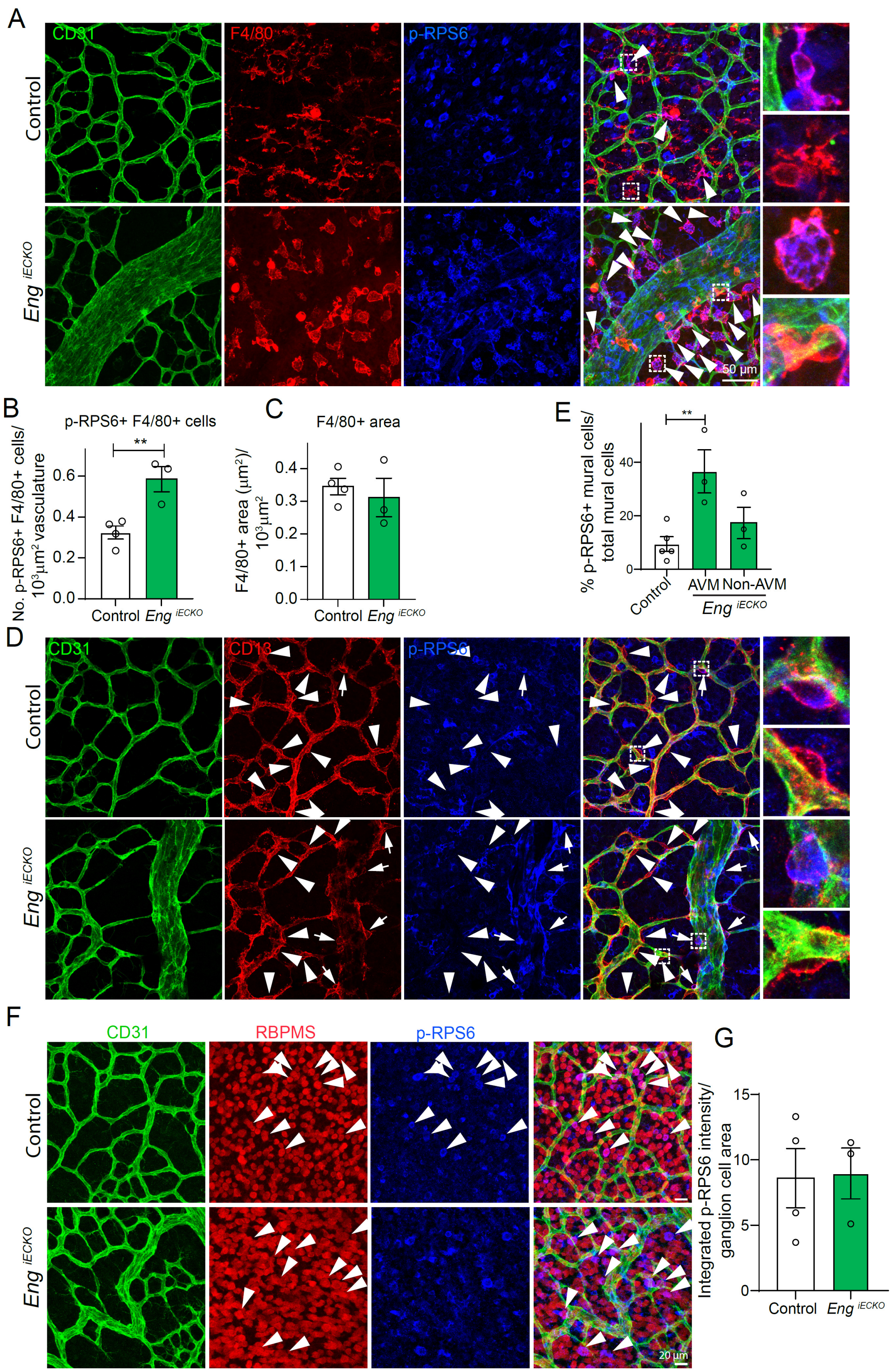

Supplementary Figure 1: ***Eng* EC deletion results in increased mTORC1 activity in macrophages, pericytes but not ganglion cells**. A. Representative images of capillary vasculature in control mouse and AVM in *Eng*^{iECKO} mouse displaying co-staining of CD31, F4/80 and p-RPS6. Examples of p-RPS6^{+} and p-RPS6^{-} cells are indicated in boxed area and shown magnified in right panels. Arrowheads indicate

F4/80$^+$ p-RPS6$^+$ cells. B. Quantification of the number of F4/80$^+$ p-RPS6$^+$ cell per vascular area in control (n=4 mice) and *Eng*^{iECKO} (n=3 mice) mice. Data analysed by two-tailed unpaired t-test with Welch's correction. C. Quantification of the F4/80$^+$ area normalized to CD31$^+$ area in control (n=4 mice) and *Eng*^{iECKO} (n=3 mice) mice. Data analysed by two-tailed unpaired t-test with Welch's correction. D. Representative images of capillary vasculature in control mouse and AVM in *Eng*^{iECKO} mouse displaying co-staining of CD31, CD13 and p-RPS6. Examples of p-RPS6$^+$ and p-RPS6$^-$ cells are indicated in boxed area and shown magnified in right panels. Arrowheads indicate CD13$^+$ p-RPS6$^-$ cells and arrows indicate CD13$^+$ p-RPS6$^+$ cells. E. Quantification of the percentage of pericytes positive for p-RPS6 in control (n=5 mice) and *Eng*^{iECKO} (n=3 mice). Data analysed by one-way ANOVA (Brown-Forsythe and Welch ANOVA tests). F. Representative images of capillary vasculature in control mouse and AVM in *Eng*^{iECKO} mouse displaying CD31, RBPMS and p-RPS6. Arrowheads indicate RBPMS$^+$ p-RPS6$^+$ cells. G. Quantification of integrated p-RPS6 intensity per ganglion cell area in control (n=4 mice) and and *Eng*^{iECKO} mice (n=3 mice). Data analysed by two-tailed unpaired t-test with Welch's correction. Bars indicate mean ± s.d.**p <0.01.

Supplementary Figure 2

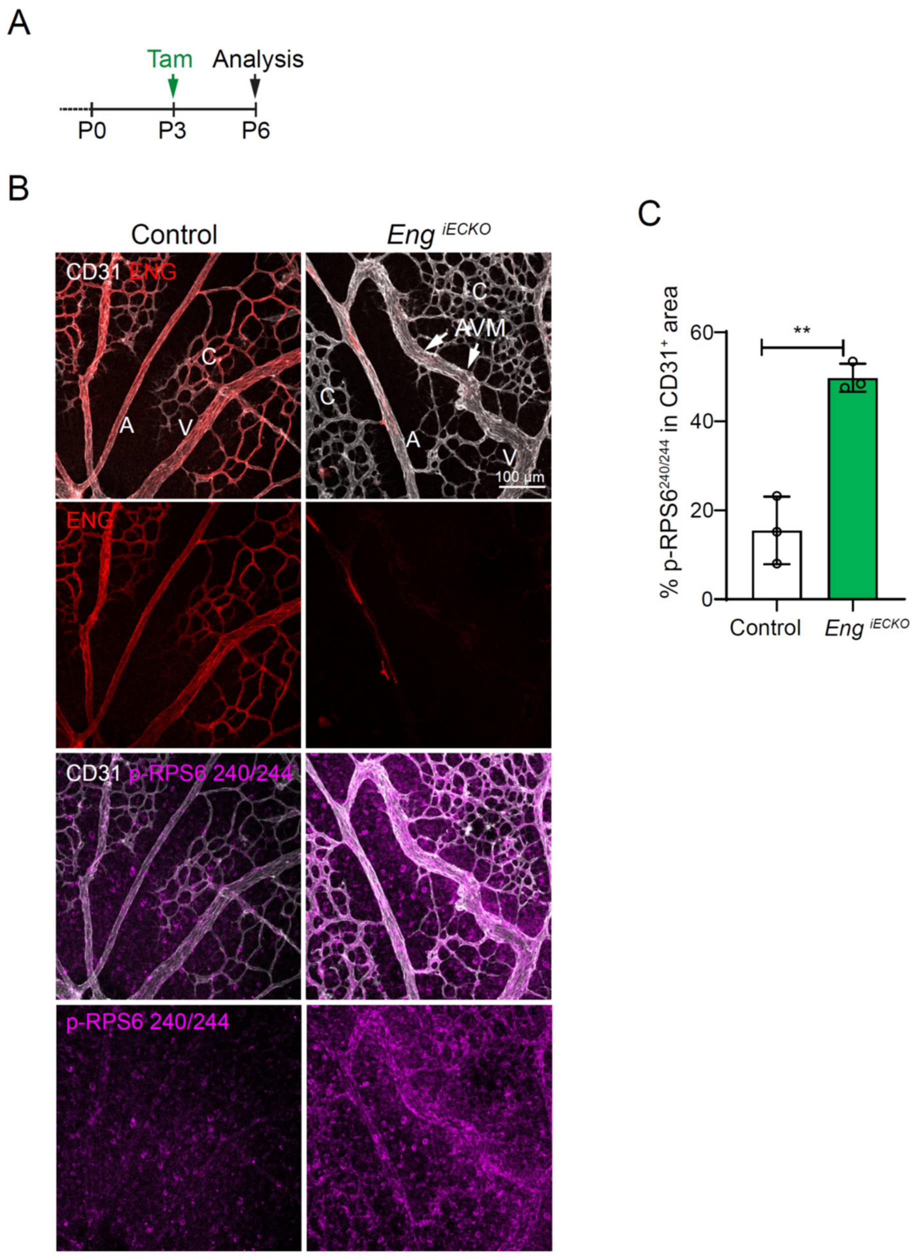

Supplementary Figure 2. EC *Eng* deletion results in increased phosphorylation of the mTORC1 specific target pS6 (240/244). A. Animals were induced by intraperitoneal injection of tamoxifen (100 µg) at P3 and retinas was harvested at P6. B. Representative images of the retinal vasculature of control and *Eng*^{iECKO} mice showing immunostaining of ENG and p-RPS6(ser240/244). C. Quantification of p-RPS6(ser240/244) levels in CD31^{+} area in the AVM region in the *Eng*^{iECKO} (n=3 mice) and a region containing artery, vein and capillaries in the control (n=3 mice). Data was analysed by two-tailed unpaired t-test with Welch's correction. Bars indicate mean ± s.d. **p<0.01.

Supplementary Figure 3

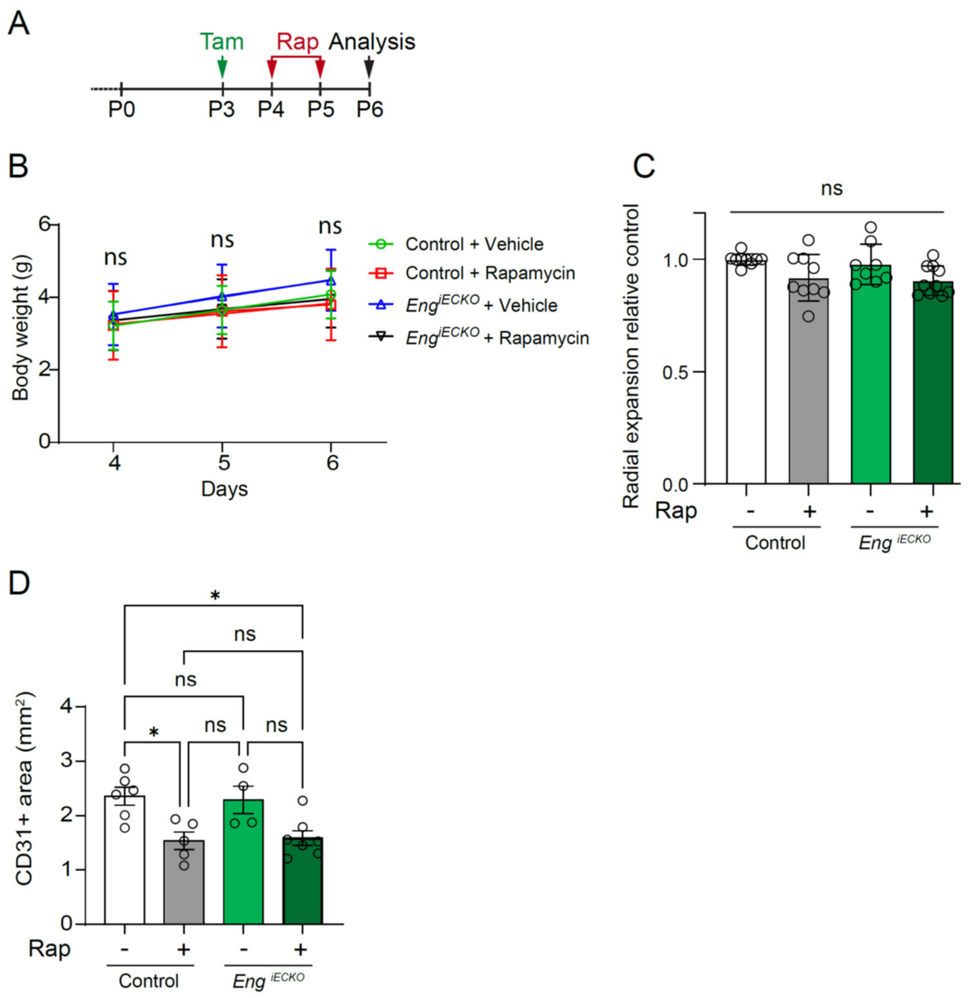

Supplementary Figure 3. Rapamycin treatment does not cause effects on body weight or radial expansion. A. Tamoxifen, vehicle and rapamycin treatment and sample collection time points in control (Cre-) and *Eng^{iECKO}* mice. B. Graphical representation of body weight of the different experimental groups over time (Control + vehicle: n=11 mice; Control + rapamycin: n=10 mice; *Eng^{iECKO}* + Vehicle: n=11 mice; *Eng^{iECKO}* + rapamycin: n=11 mice). Data was analysed by two-way ANOVA. C. Quantification of the radial expansion in the different experimental groups (Control + vehicle: n=9 mice; Control + rapamycin: n=9 mice; *Eng^{iECKO}* + Vehicle: n=8 mice; *Eng^{iECKO}* + rapamycin: n=10 mice). Data was analysed by one-way ANOVA (Brown-Forsythe and Welch ANOVA tests). D. Quantification of the vascular density in the different experimental groups (Control + vehicle: n=6 mice; Control + rapamycin: n=5 mice; *Eng^{iECKO}* + Vehicle: n=4 mice; *Eng^{iECKO}* + rapamycin: n=7 mice). Data was analysed by one-way ANOVA (Brown-Forsythe and Welch ANOVA tests). Bars indicate mean ± s.d. *p-value<0.05; n.s. indicates no significant difference.

Supplementary Figure 4

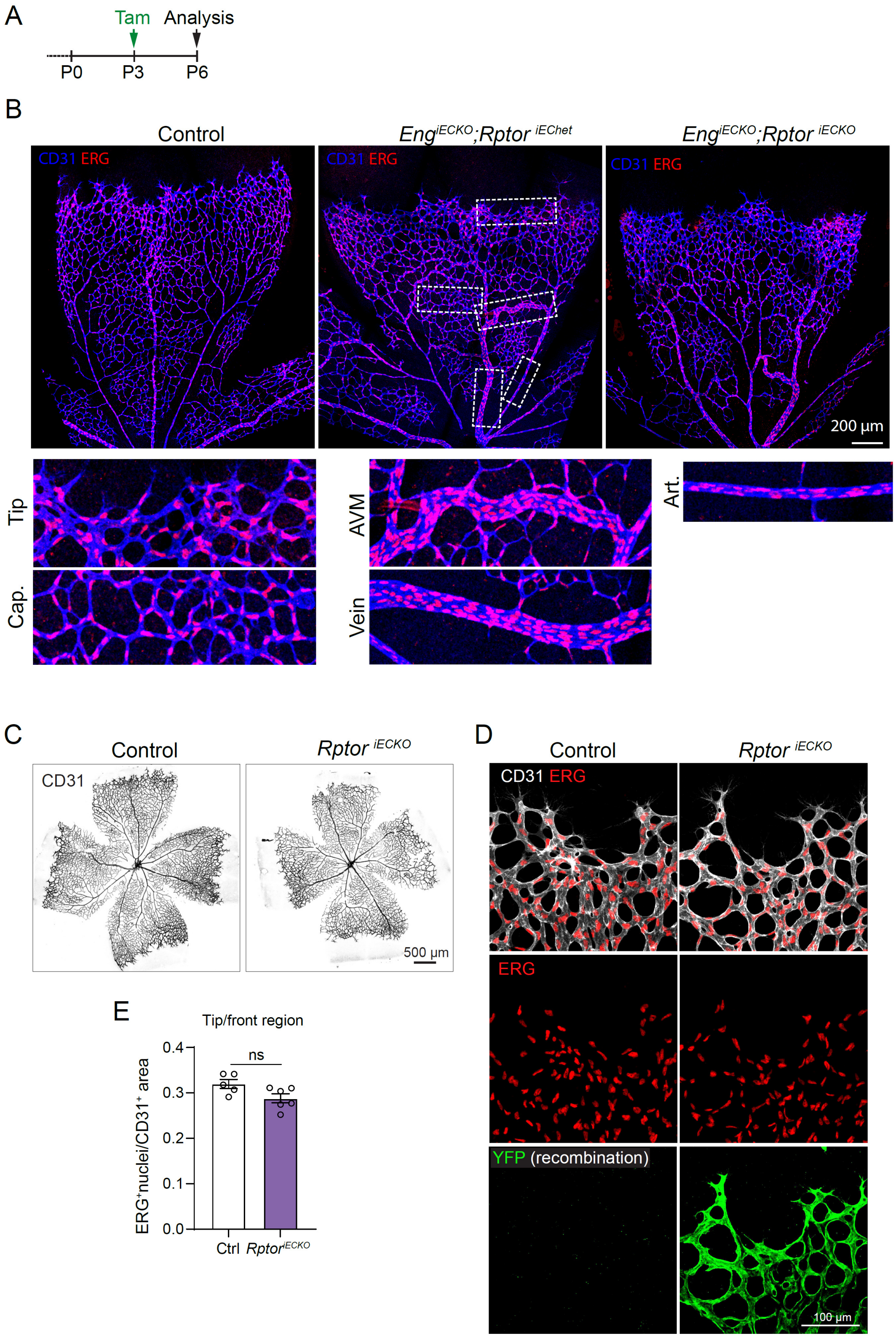

Supplementary Figure 4. EC genetic inactivation of mTORC1 reduces vascular growth and affects EC number. A. Tamoxifen treatment (P3) and sample collection time point (P6) for postnatal EC-specific deletion of *Eng* and *Rptor* in *Eng*^{iECKO} *Rptor*^{iECKO} and *Rptor* in *Rptor*^{iECKO}. B. Representative pictures of

Control, *Eng*^{iECKO}: *Rptor*^{iEChet} , *Eng*^{iECKO}: *Rptor*^{iECKO} vasculature displaying co-staining of CD31 and ERG. Boxed boxed areas from *Eng*^{iECKO}: *Rptor*^{iEChet} are shown magnified in the panels below. C. Representative pictures of retinal vasculature of Control and *Rptor*^{iECKO} illustrated by CD31 staining. D. Representative pictures of the sprouting front of Control and *Rptor*^{iECKO} retinas showing immunostaining of CD31, ERG and YFP. E. Quantification of the number of ERG^{+} cells per CD31 area of Control (n= 5 mice) and *Rptor*^{iECKO} mice (n= 6 mice). Data was analysed by two-tailed unpaired t-test with Welch's correction. Bars indicate mean ± s.d. ns, no significant difference.

Supplementary Figure 5

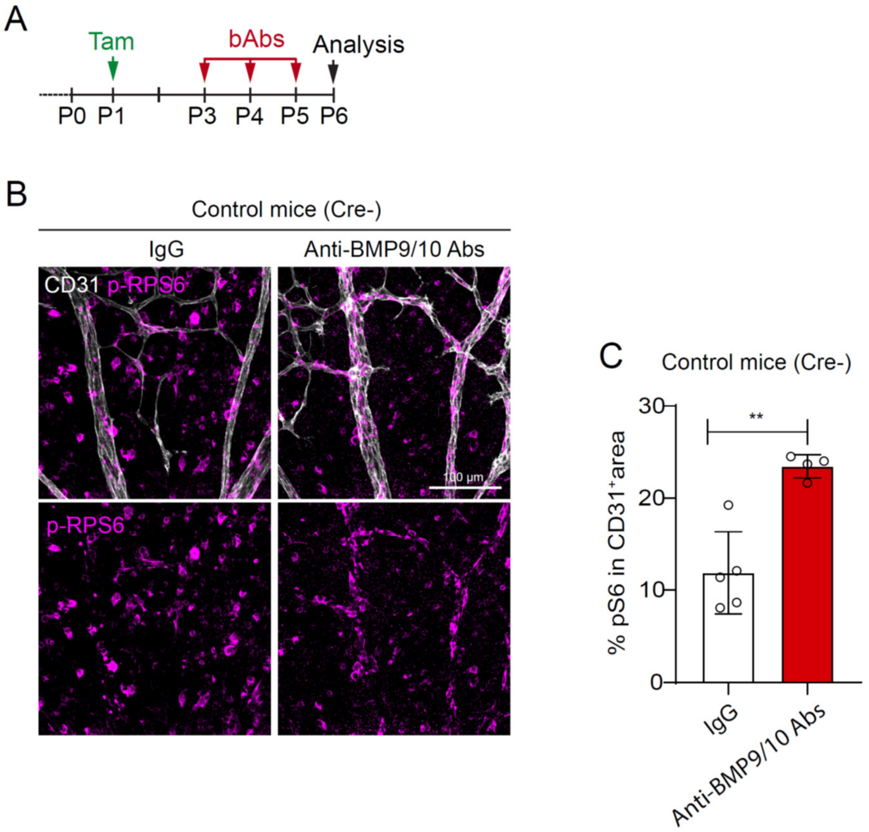

Supplementary Figure 5: AVMs induced by blocking antibodies displayed higher p-RPS6 levels compared to the control vasculature. A. Time scheme for administration of tamoxifen, BMP9/10 blocking antibodies (bAbs)/Isotype IgG, and sample collection. B. Representative pictures of vasculature in mice treated with isotype IgG and AVM in mice treated with BMP9/10 blocking antibodies. C. Quantification of p-RPS6 levels in CD31+ area in the vascular plexus of control IgG (n=5 mice) and anti-BMP9/10 (n=4 mice) treated mice. Data analysed by two-tailed unpaired t-test with Welch's correction. Bars indicate mean ± s.d. **p<0.01.
